# Supplementary material for: Use of chitin:DNA ratio to assess growth form in fungal cells
Source: BMC Biol. 2024 Jan 17;22:10. doi: 10.1186/s12915-024-01815-2 (PMC10795418; doi:10.1186/s12915-024-01815-2)
Supplement: Supplementary file 1 — Additional file 1: Figure S1. Effect of leaf health on background fluorescence. We carried out chitin quantitation assays on samples of inoculated leaf material immediately after inoculation. For inoculation we selected leaves which could easily be visually distinguished on the basis of colour. Combined with the inclusion of the ‘no dye’ controls for each sample, this allowed us to determine whether apparently healthy green leaves provided a different level of background fluorescence than senescing, yellow leaves. Fluorescence values from yellow leaves were consistently higher. Values are means of 4 replicates and error bars show SE. [file 12915_2024_1815_MOESM1_ESM.docx]

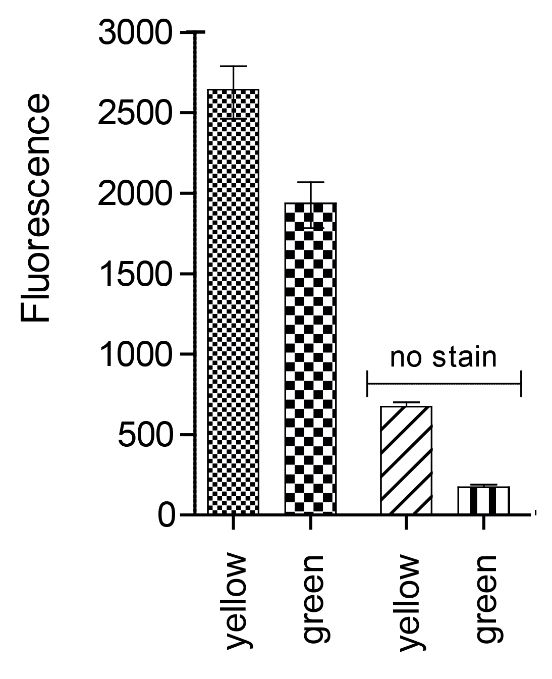


**Figure S1: Effect of leaf health on background fluorescence.** We carried out chitin quantitation assays on samples of inoculated leaf material immediately after inoculation. For inoculation we selected leaves which could easily be visually distinguished on the basis of colour. Combined with the inclusion of the ‘no dye’ controls for each sample, this allowed us to determine whether apparently healthy green leaves provided a different level of background fluorescence than senescing, yellow leaves. Fluorescence values from yellow leaves were consistently higher. Values are means of 4 replicates and error bars show SE.
