# Supplementary material for: Use of chitin:DNA ratio to assess growth form in fungal cells
Source: BMC Biol. 2024 Jan 17;22:10. doi: 10.1186/s12915-024-01815-2 (PMC10795418; doi:10.1186/s12915-024-01815-2)
Supplement: Supplementary file 2 — Additional file 2: Figure S2. Autoclave vs heatblock during chitin quantitation. We compared the FITC-fluorescence readings for samples processed by autoclaving, as in the original method of Ayliffe et al. (2013) [24] vsthe use of a heatblock, which provides some practical advantages when working with large numbers of samples. The heatblock method appears to be better able to liberate chitin for WGA-FITC binding when larger amounts are present in the sample. Values are means of 4 replicates and error bars show SE. [file 12915_2024_1815_MOESM2_ESM.docx]

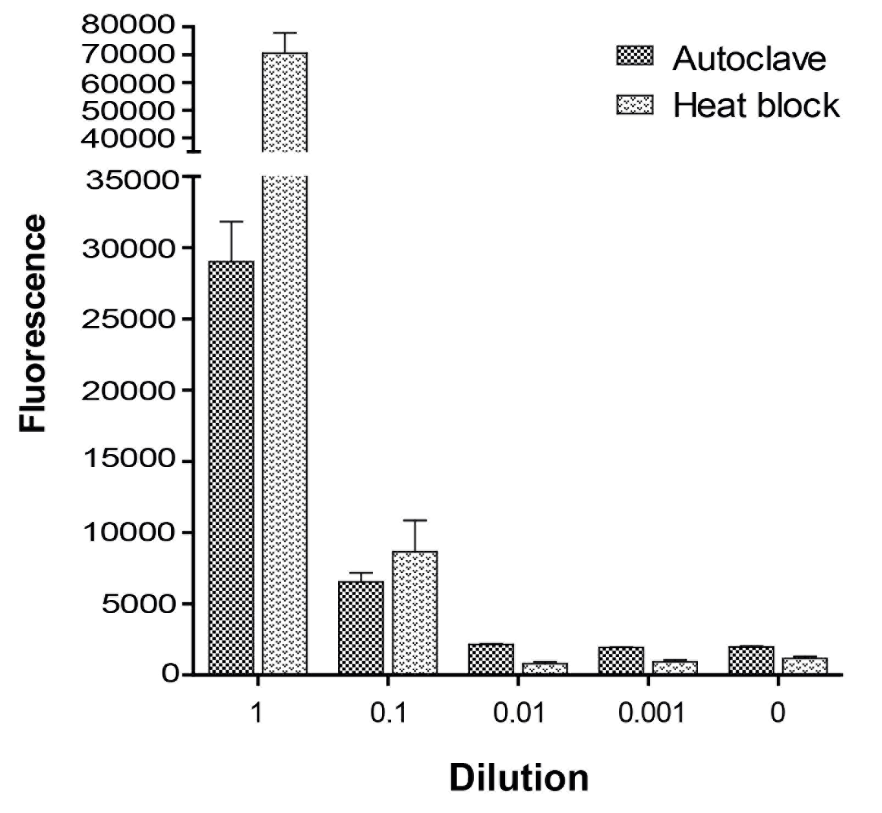


**Figure S2: Autoclave vs heatblock during chitin quantitation.**  We compared the FITC-fluorescence readings for samples processed by autoclaving, as in the original method of Ayliffe *et al.* (2006) *vs* the use of a heatblock, which provides some practical advantages when working with large numbers of samples. The heatblock method appears to be better able to liberate chitin for WGA-FITC binding when larger amounts are present in the sample. Values are means of 4 replicates and error bars show SE.
