# Supplementary material for: Use of chitin:DNA ratio to assess growth form in fungal cells
Source: BMC Biol. 2024 Jan 17;22:10. doi: 10.1186/s12915-024-01815-2 (PMC10795418; doi:10.1186/s12915-024-01815-2)
Supplement: Supplementary file 3 — Additional file 3: Figure S3. Leaf vs cellulose as carrier. We tested the use of both uninfected leaf material and purified cellulose as‘carriers’ for small samples (10 or 20 mg) of in vitro grown fungus during the homogenisation step. Both sets of results show the expected increase in fluorescence when more fungus is present in the original sample. Values are means of 4 replicates and error bars show SE. [file 12915_2024_1815_MOESM3_ESM.docx]

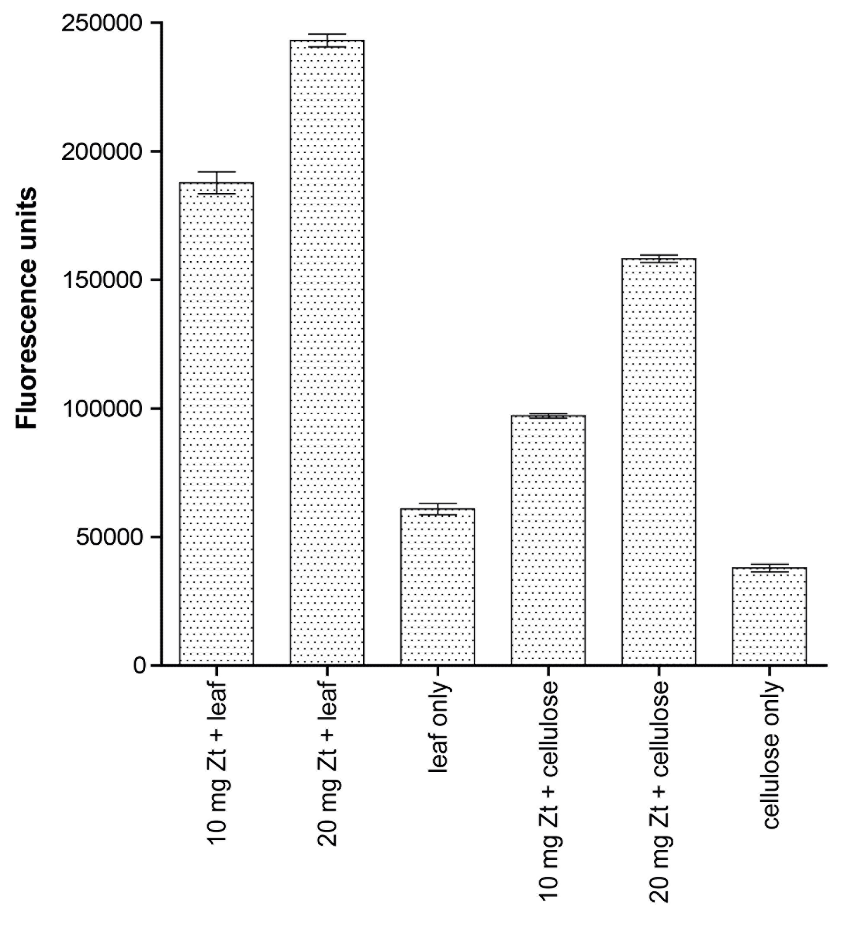


**Figure S3: Leaf vs cellulose as carrier.**  We tested the use of both uninfected leaf material and purified cellulose as ‘carriers’ for small samples (10 or 20 mg) of *in vitro* grown fungus during the homogenisation step. Both sets of results show the expected increase in fluorescence when more fungus is present in the original sample. Values are means of 4 replicates and error bars show SE.
